# Case report: Identification of novel *fibrillin-2* variants impacting disulfide bond and causing congenital contractural arachnodactyly

**DOI:** 10.3389/fgene.2023.1035887

**Published:** 2023-03-03

**Authors:** An-Lei Li, Ji-Qiang He, Lei Zeng, Yi-Qiao Hu, Min Wang, Jie-Yi Long, Si-Hua Chang, Jie-Yuan Jin, Rong Xiang

**Affiliations:** ^1^ Department of Orthopaedics, Xiangya Hospital of Central South University, Changsha, China; ^2^ School of Life Sciences, Central South University, Changsha, China; ^3^ National Clinical Research Center for Geriatric Disorders, Xiangya Hospital of Central South University, Changsha, China; ^4^ Hunan Key Laboratory of Animal Models for Human Diseases, School of Life Sciences, Central South University, Changsha, China; ^5^ Hunan Key Laboratory of Medical Genetics, School of Life Sciences, Central South University, Changsha, China; ^6^ Department of Nephrology, Xiangya Hospital of Central South University, Changsha, China

**Keywords:** FBN2, congenital contractural arachnodactyly, arthrogryposis, disulfide bond, whole-exome sequencing

## Abstract

**Background:** Congenital contractural arachnodactyly (CCA) is an autosomal dominant connective tissue disorder with clinical features of arthrogryposis, arachnodactyly, crumpled ears, scoliosis, and muscular hypoplasia. The heterozygous pathogenic variants in *FBN2* have been shown to cause CCA. Fibrillin-2 is related to the elasticity of the tissue and has been demonstrated to play an important role in the constitution of extracellular microfibrils in elastic fibers, providing strength and flexibility to the connective tissue that sustains the body’s joints and organs.

**Methods:** We recruited two Chinese families with arachnodactyly and bilateral arthrogryposis of the fingers. Whole-exome sequencing (WES) and co-segregation analysis were employed to identify their genetic etiologies. Three-dimensional protein models were used to analyze the pathogenic mechanism of the identified variants.

**Results:** We have reported two CCA families and identified two novel missense variants in *FBN2* (NM_001999.3: c.4093T>C, p.C1365R and c.2384G>T, p.C795F). The structural models of the mutant FBN2 protein in rats exhibited that both the variants could break disulfide bonds.

**Conclusion:** We detected two *FBN2* variants in two families with CCA. Our description expands the genetic profile of CCA and emphasizes the pathogenicity of disulfide bond disruption in FBN2.

## Introduction

Congenital contractural arachnodactyly (CCA; OMIM 121050) was first described in 1968, and Beals and Hecht (1971) later seceded it from the Marfan syndrome (MFS; OMIM 154700), thus the naming of CCA as also the Beals–Hecht syndrome (Callewaert et al., 1993). The incidence of CCA is low, but given that its symptoms overlap with MFS, the actual percentage is hard to estimate (Tuncbilek and Alanay, 2006). CCA is a connective tissue disease. People with CCA share many distinguishing features, such as arachnodactyly, camptodactyly, crumpled ears, scoliosis, pectus deformities, flexion contractures of multiple joints (especially, fingers, elbows, and knee joints), and muscular hypoplasia (Qiu et al., 2021; Sun et al., 2022). CCA has phenotypic heterogeneity, the phenotypes of which can vary within and between families. In the most severe type, “severe CCA with cardiovascular and/or gastrointestinal anomalies”—a rare phenotype in infants with pronounced features of CCA (severe crumpling of the ears, arachnodactyly, contractures, congenital scoliosis, and/or hypotonia) and severe cardiovascular and/or gastrointestinal anomalies—it exhibits no specific geographic or ethnic predilection (Frederic et al., 2009).

CCA is caused by variants in *FBN2* located on 5q23-31. *FBN2* encodes fibrillin-2, a big secretory protein containing 2,912 amino acids that are expressed during early embryonic development (Peeters et al., 2021). FBN2 is related to the elasticity of the connective tissue and has been demonstrated to play an important role in the constitution of extracellular microfibrils in elastic fibers, providing strength and flexibility to the connective tissue that sustains the body’s joints and organs (Shi et al., 2013). There are other diseases that are triggered by *FBN2* variants, namely, the Marfan syndrome with aortopathy, talipes equinovarus, macular degeneration, and scoliosis (Ansari et al., 2014; Buchan et al., 2014; Ratnapriya et al., 2014; Du et al., 2021).

In this study, we identified two novel *FBN2* variants (NM_001999.3: c.4093T>C, p.C1365R and c.2384G>T, p.C795F) in two Chinese families diagnosed with CCA, which are likely the underlying etiologies in these two families.

## Materials and methods

### Patients and subjects

**FIGURE 1:**
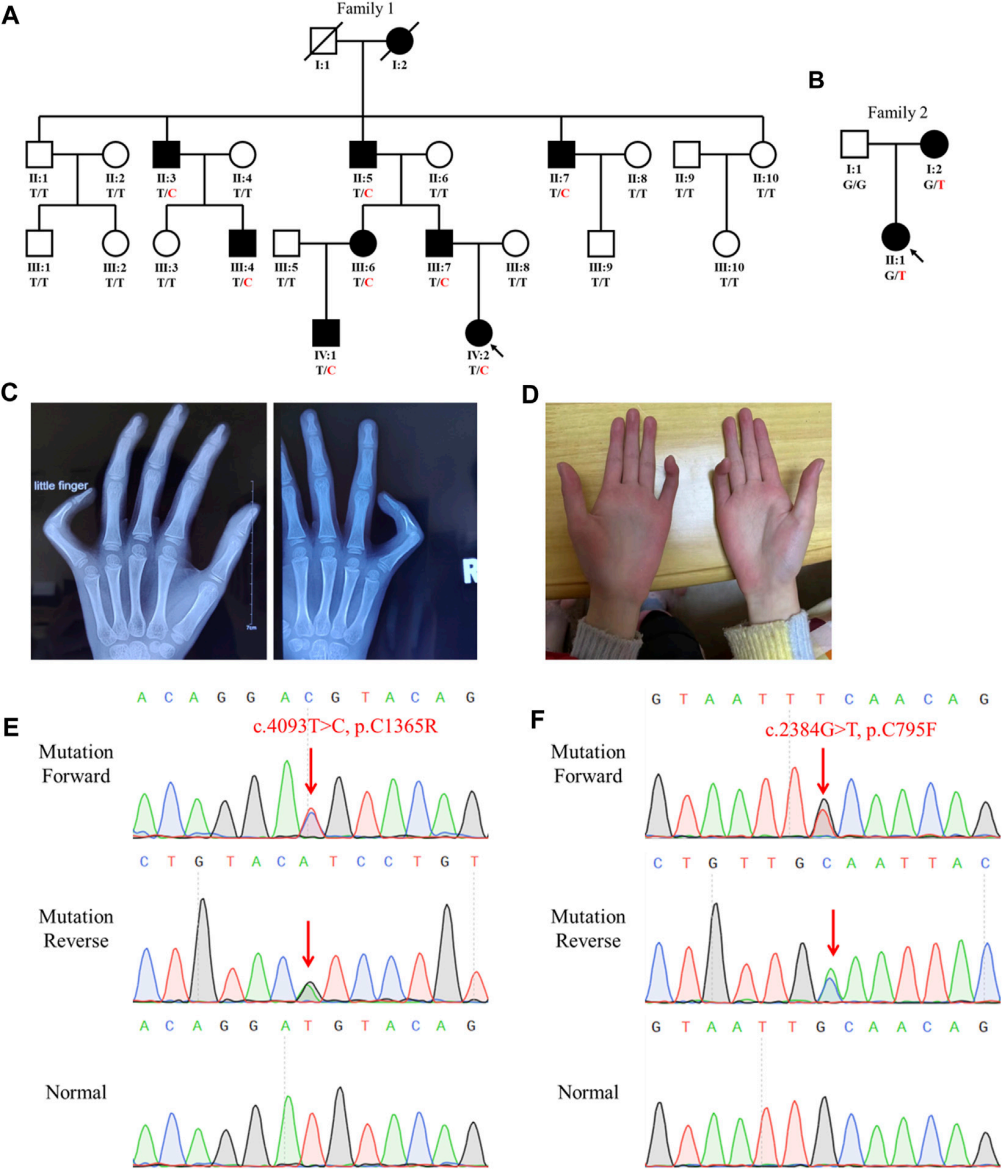
Family genogram, symptoms, and Sanger sequencing of the probands. **(A,B)** Genogram of the F1 family and F2 family. Black symbols represent the affected members, slashes indicate those who died, and the arrow indicates the proband. All recruited subjects underwent genetic testing, and their genotypes are identified by letters and a slash, with red representing variants. Question marks represent unknown data or symptoms. **(C,D)** F1 family proband had arachnodactyly and bilateral contracture of the fifth fingers. **(E,F)** Sequencing results of *FBN2* variants using Sanger sequencing. The red arrow points to the variant site.

This research was approved by the Review Board of Xiangya Hospital of the Central South University (approval number: 202103427). We recruited two CCA families (Figures 1A,B). Written informed consent was obtained from all subjects. All patients or their guardians consented to publish the clinical details. Two milliliters of blood per participant was collected and the genomic DNA was extracted using the DNeasy Blood & Tissue Kit (Qiagen, Valencia, United States) for all subjects.

### Whole-exome sequencing

Berry Genomics Company Limited (Chengdu, China) performed the exome capture, high-throughput sequencing, and common filtering. One milligram of genomic DNA was randomly carved using a Covaris S220 sonicator (Covaris, Inc., Woburn, United States). The fragmented DNA was subjected to three enzymatic steps: end repair, A-tailing, and adapter ligation. It was amplified with Herculase II Fusion DNA Polymerase (Agilent Technologies, Inc., Santa Clara, United States). The exomes in the pre-capture libraries were captured by the SureSelect capture library kit (Agilent Technologies, Inc., Santa Clara, United States). The captured DNA library was then used for the next-generation sequencing on the Illumina HiSeq 4000 platform (Illumina Inc., San Diego, United States). Downstream processing was carried out using the Genome Analysis Toolkit (GATK), VarScan 2, and Picard, and the variant calls were made using the GATK HaplotypeCaller. Variant annotation referred to Ensembl release 82, and filtering was conducted using the ANNOVAR documentation.

We annotated the variants’ frequencies on the basis of the data from the Genome Aggregation Database (GnomAD; http://gnomad.broadinstitule.org) and 1000 Genomes Project (1000G; https://www.internationalgenome.org/) and predicted their pathogenicity using the MutationTaster (http://www.mutationtaster.org/), PolyPhen-2 (http://genetics.bwh.harvard.edu/pph2/), SIFT (http://provean.jcvi.org/index.php), and Combined Annotation–Dependent Depletion (CADD; https://cadd.gs.washington.edu/snv; variants with scores greater than 15 were considered as pathogenic). The variants were retained if they met the following two conditions: 1) the frequency of the variant was less than 0.001 and 2) at least three bioinformatics software predicted the variant to be causative. These variants were filtered when they were against the skeletal dysplasia–related genes (Supplementary Table S1). For selected variants, the inheritance patterns and clinical phenotypes of the genes were annotated using OMIM (https://www.omim.org) and the American College of Medical Genetics and Genomics (ACMG) Standards and Guidelines was used to classify their pathogenicity grade (Richards et al., 2015).

### Sanger sequencing

The *FBN2* reference sequence and transcript sequence (NM_001999.3) were acquired from the NCBI (https://www.ncbi.nlm.nih.gov/gene/2201). The primer pairs used in *FBN2* variant verification (FBN2 f1: 5′-AGA​TCT​TGC​CTG​TGT​ATT​TCA​GTA-3′, FBN2 r1: 5′-GCT​CTA​AGA​GAT​GCC​AGA​GAA​C-3′, annealing temperature: 57.2°C; FBN2 f2: 5′-GAA​TTT​CTG​CCA​GCG​TCT​TTC-3′, FBN2 r2: 5′-GTA​CCA​CCA​AGA​AGT​CTG​TTA​CT-3′, annealing temperature: 57.8°C) were designed by Integrated DNA Technologies (https://sg.idtdna.com/Primerquest/Home/Index). The polymerase chain reaction (PCR) system, which included 12.5 μL 2× PCR mix, 11 μL ultra-pure water, 0.5 μL DNA sample, 0.5 μL and 10 μM forward primer, and 0.5 μL and 10 μM reverse primer, was operated using the Biometra TOne 96G PCR amplifier (Analytik Jena, Jena, Germany). The running program was divided into four stages: 1) 95°C for 5 min; 2) sustaining 35 cycles as 95°C for 30 s, annealing temperature for 30 s, and 72°C for 1 min; 3) 72°C for 10 min; and 4) maintaining 4°C for storing the samples. The PCR products were sequenced by Sangon Biotech Company Limited (Shanghai, China).

### Mutant protein modeling

The FBN2 protein structure of rats (F1M5Q4) was downloaded from the AlphaFold Protein Structure Database (https://alphafold.ebi.ac.uk/entry/F1M5Q4). The amino acid sequences used in the homology comparison of FBN2 were obtained from the NCBI (https://www.ncbi.nlm.nih.gov/protein/?term=FBN2). PyMOL was used to construct rat mutant FBN2 models based on the wild-type structure.

## Results

### Case description

The proband (IV:2) of the F1 family was a six-year-old girl from Hunan province, China (Figure 1A). She was admitted to our hospital for her contractures of bilateral fifth fingers (Figures 1C, D). She was tall (1.3 m, >0.95%) and had slender limbs, arachnodactyly, and crumpled ears (Figure 1D). Except for the abovementioned symptoms, the proband did not present other phenotypes. We thus diagnosed her with CCA. Tracing back her family history, her father (III:7) and seven family members (I:2, II:3, II:5, II:7, III:4, III:6, and IV:1) also exhibited similar phenotypes, namely, a tall and slender look, flexion contractures of proximal interphalangeal joints, and crumpled ears (Table 1). Her mother (III:8) and other subjects (II:1, II:2, II:4, II:6, II:8, II:9, II:10, III:1, III:2, III:3, III:5, III:8, III:9, and III:10) were unaffected.

**TABLE 1:** Phenotypes of those affected in the F1 family.

Patients	Gender	Age (years)	Height (cm)	Arachnodactyly	Arthrogryposis	Crumpled ears	Others
I:2	F	78	Unknown	Unknown	Contractures of bilateral fifth fingers	Unknown	SCD
II:3	M	63	178	+	Contractures of bilateral fifth fingers	−	Coronary heart disease
II:5	M	58	183	+	−	+	Unknown
II:7	M	55	180	+	Contractures of bilateral fifth fingers	−	Hypertension
III:4	M	37	181	+	Contractures of bilateral fifth fingers	Unknown	Unknown
III:6	F	34	178	+	Contractures of bilateral fifth fingers	+	—
III:7	M	32	182	+	Contractures of bilateral fifth fingers	−	—
IV:1	M	8	134	+	Contractures of bilateral fifth fingers	+	—
IV:2	F	6	130	+	Contractures of bilateral fifth fingers	+	—

M, male; F, female; +, positive phenotype; −, negative phenotype; SCD, sudden cardiac death.

The proband (II:1) of the F2 family was a one-year-old girl. Both her mother and she exhibited arthrogryposis of bilateral third fingers and I:2 had arachnodactyly. Her father (I:1) was unaffected.

### Genetic analysis

We extracted the genomic DNA of the probands’ peripheral blood and operated whole-exome sequencing (WES). We eliminated the variants with frequencies over 0.001 in GnomAD and 1000G or a prediction of benign by the MutationTaster, PolyPhen-2, SIFT, and CADD. The rest of the variants were filtered against the skeletal dysplasia–related genes (Supplementary Table S1). Finally, six variants were identified in the F1 family proband and three variants in the F2 family proband (Table 2). Adhering to the standards and guidelines of the ACMG, we classified these two *FBN2* variants (NM_001999.3: c.4093T>C, p.C1365R and c.2384G>T, p.C795F) as “Likely pathogenic.”

**TABLE 2:** Variants identified in the two probands by WES in combination with the filtration of skeletal dysplasia–related genes.

Gene	Variant	Pathogenicity prediction	GnomAD	1000G		OMIM clinical phenotype	American College of Medical Genetics classification
**F1 family proband**							
*DYNC2H1*	NM_001080463.1: c.10073A>G, p.Y3358C	MutationTaster: D	0.00000	—		AD/AR, short-rib thoracic dysplasia 3 with or without polydactyly	Uncertain significance (PM2, PP3, and BS4)
		PolyPhen-2: D					
		SIFT: D					
		CADD: 27					
*GNPTAB*	NM_024312.4: c.1091G>A, p.R364Q	MutationTaster: D	0.00006	—		AR, mucolipidosis II alpha/beta; AR, mucolipidosis III alpha/beta	Uncertain significance (PM2, PP3, and BS4)
		PolyPhen-2: D					
		SIFT: D					
		CADD: 32					
*KIAA0586*	NM_001244189.1: c.3950A>T, p.D1317V	MutationTaster: D	—	—		AR, Joubert syndrome 23; AR, short-rib thoracic dysplasia 14 with polydactyly	Uncertain significance (PM2, PP3, and BS4)
		PolyPhen-2: P					
		SIFT: D					
		CADD: 20					
*MKS1*	NM_017777.3: c.199C>T, p.R67C	MutationTaster: D	0.00012	—		AR, Bardet–Biedl syndrome 13; AR, Joubert syndrome 28; AR, Meckel syndrome 1	Uncertain significance (PM2, PP3, and BS4)
		PolyPhen-2: P					
		SIFT: D					
		CADD: 24					
*LIFR*	NM_002310.5: c.247A>T, p.I83F	MutationTaster: D	—	—		AR, Stuve–Wiedemann syndrome/Schwartz–Jampel type 2 syndrome	Uncertain significance (PM2, PP3, and BS4)
		PolyPhen-2: B					
		SIFT: D					
		CADD: 17					
*FBN2*	NM_001999.3: c.4093T>C, p.C1365R	MutationTaster: D	—	—		AD, contractural arachnodactyly, congenital; AD, macular degeneration, early onset	Likely pathogenic (PM1, PM2, PP1, PP3, and PP4)
		PolyPhen-2: D					
		SIFT: D					
		CADD: 30					
**F2 family proband**							
*FBN2*	NM_001999.3: c.2384G>T, p.C795F	MutationTaster: D	—	—		AD, contractural arachnodactyly, congenital; AD, macular degeneration, early onset	Likely pathogenic (PM1, PM2, PP1, and PP3)
		PolyPhen-2: D					
		SIFT: D					
		CADD: 29					
*COL1A1*	NM_000088.3: c.3766G>A, p.A1256T	MutationTaster: D	0.00023	0.00100		AD, Caffey disease; AD, combined osteogenesis imperfecta and Ehlers–Danlos syndrome 1; AD, Ehlers–Danlos syndrome, arthrochalasia type, 1; AD, osteogenesis imperfecta, type I–IV	Uncertain significance (PS1, PP3, and BS4)
		PolyPhen-2: P					
		SIFT: D					
		CADD: 24					
*COL6A1*	NM_001848.2: c.1898T>C, p.I633T	MutationTaster: D	0.00001	—		AD/AR, Bethlem myopathy 1; AD/AR, Ullrich congenital muscular dystrophy 1	Uncertain significance (PM2, PP3, and BS4)
		PolyPhen-2: P					
		SIFT: D					
		CADD: 26					

D, disease-causing; P, polymorphism; B, benign; N, natural; AR, recessive dominant; AD, autosomal dominant.

**FIGURE 2:**
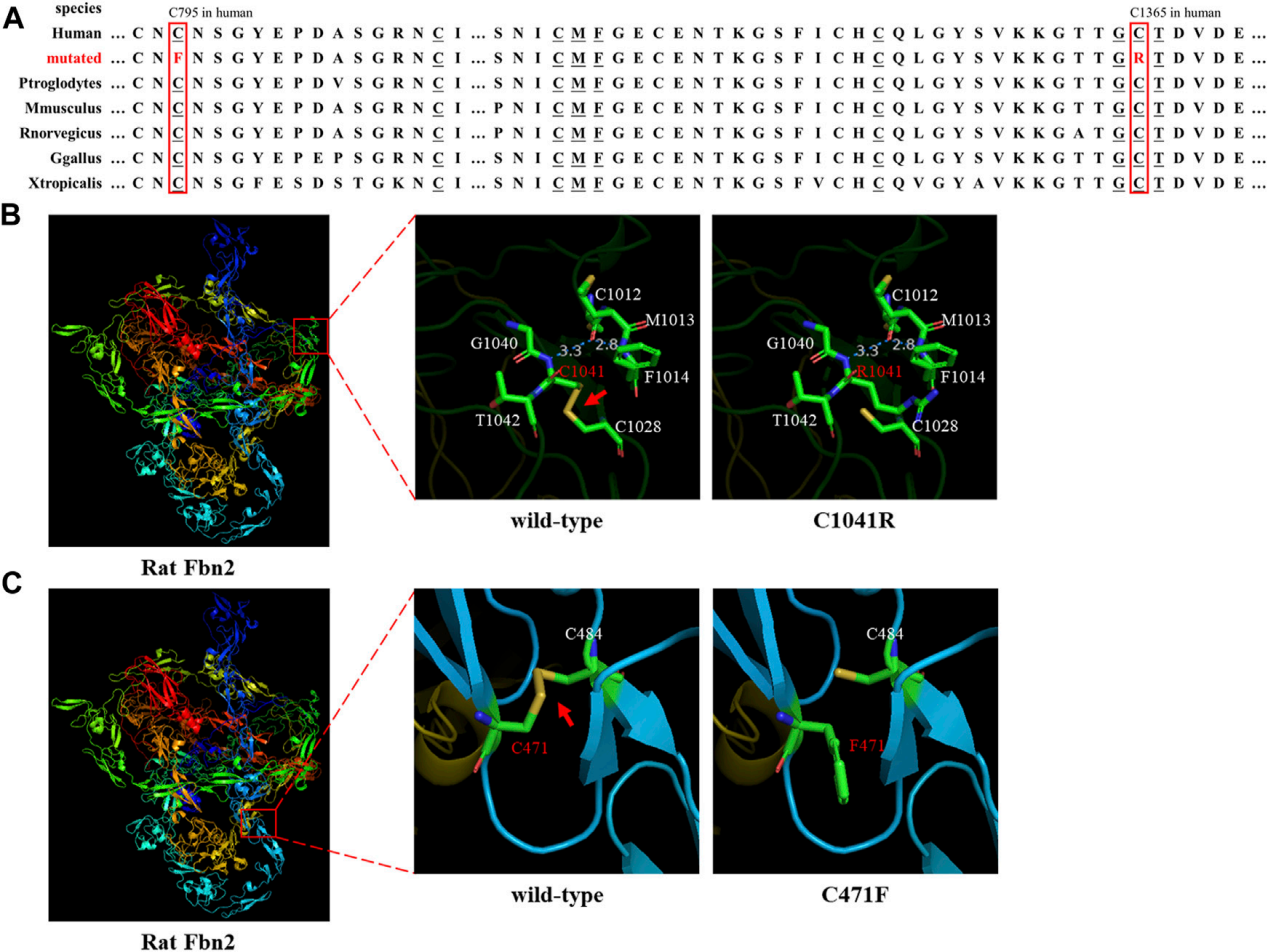
Species conservation analysis and protein modeling. **(A)** Species conservation analysis of the mutant amino acid sites of FBN2. The red box represents the mutant amino acid site in various species. Underlined amino acids correspond to those labeled in Figures 2B,C that are highly evolutionarily conserved. **(B,C)** Three-dimensional model of rat FBN2 with wild type or p.C1041R/p.C471 variant. The red arrow points to the disulfide bond.

Sanger sequencing showed that the *FBN2* variant c.4093T>C in the F1 proband was inherited from her father, and all patients harbored this variant (Figures 1A,E). Another *FBN2* variant, c.2384G>T, was identified in the F2 proband and her mother (Figures 1B,F). The amino acid sequence alignment analysis indicated that p.C795 and p.C1365 in FBN2 were highly conserved throughout evolution (Figure 2A). According to the three-dimensional modeling of the FBN2 protein in rats, these amino acid alterations broke the disulfide bonds and may have triggered instability of the FBN2 structure, and these two regions are homologous in humans and rats (Figure 2). Thus, we reasoned that the *FBN2* variants c.4093T>C, p.C1365R and c.2384G>T, p.C795F were the genetic etiologies of the F1 and F2 families, respectively.

## Discussion

Patients with CCA or MFS share many distinguishing features, such as the so-called marfanoid appearance, that is, a tall, slender, and asthenic appearance and skeletal features (Najafi et al., 2020). In contrast with MFS, most individuals with CCA have crumpled ears, flexion contractures, muscular hypoplasia, and no ocular and cardiovascular complications (Lavillaureix et al., 2017). Initially, the F1 proband was admitted to our hospital for contractures of her bilateral fifth fingers, and then we noticed her classical marfanoid appearance, that is, arachnodactyly, camptodactyly, and tall slender build (Sun et al., 2022). Given that the proband exhibited crumpled ears, without a family medical history of typical MFS-related cardiovascular diseases, we made a presumptive diagnosis of CCA. The F2 proband was a baby, and we did not do a full check and only determined her arthrogryposis.

To further determine their disorders, we performed molecular diagnosis by WES and detected a missense variant (c.4093T>C, p.C1365R) in exon 31 of *FBN2* in the F1 proband. There were nine patients in this family, and Sanger sequencing showed that the variant was only identified in the affected members. In accordance with the ACMG guidelines, we determined the pathogenicity classification of the *FBN2* variant as “Likely pathogenic”: 1) the variant replaced cysteine with arginine, breaking the disulfide bonds and destabilizing the protein structure (PM1); 2) the variant was absent from the controls in GnomAD and 1000G (PM2); 3) in the F1 family, only the *FBN2* variant carriers presented with CCA (PP1); given that the family included nine patients across four generations and 22 living members had participated in the present study, the evidence can be considered as strong evidence of pathogenicity; 4) The MutationTaster, PolyPhen-2, SIFT, and CADD all predicted that the variant was disease-causing (PP3); and 5) the symptoms in this family suggested a suspected case of MFS or CCA and followed an autosomal dominant inheritance pattern, suggesting a monogenic disease (PP4) (Miller et al., 2021). We considered that the *FBN2* variant was responsible for the disease of the F1 family and made a diagnosis of CCA in this family.

Another *FBN2* variant (c.2384G>T, p.C795F) in this study that was identified in the F2 family was also classified as “Likely pathogenic” on the basis of the pathogenicity category of PM1, PM2, PP1, and PP3, as per the ACMG guidelines: 1) similar to p.C1365R, variant p.C795F also destroyed the disulfide bond, maybe destabilizing the protein structure—with many known FBN2 variants also causing the destruction of disulfide bonds (PM1); 2) the variant was not listed in the GnomAD and 1000G databases (PM2); 3) only the proband and her mother presented with symptoms in this family and harbored the variant, and the genotype and phenotype were co-segregated in this family (PP1); and 4) the bioinformatics software (MutationTaster, PolyPhen-2, SIFT, and CADD) predicted the “disease-causing” nature of this variant (PP3). The molecular diagnosis and phenotype of the mother (F2 I:2) prompted that it was a CCA family.

*FBN2* contains 65 exons and codes fibrillin-2 (Kloth et al., 2021). FBN2 contains a 4-cysteine motif, 47 EGF-like domains (43 EGF-like domains with conserved calcium-binding consensus sequences called cb-EGF-like domains), two hybrid domains, seven TGF-binding proteins (TGFBP)–like modules, a glycine-rich domain, and a domain presenting with the homology to fibulins C-terminal domain III (FibuCTDIII-like motif) (Ratnapriya et al., 2014; Yin et al., 2019). In each cb-EGF-like domain, six conserved cysteine residues form three disulfide bridges for the purpose of protein stability (Davis and Summers, 2012). Missense variants can directly change the cb-EGF-like domain in the FBN2 protein and affect the constitution of the extracellular matrix microfibers. It has been shown in the following three ways: 1) variants change the Cys residue number in the cb-EGF-like domain and influence the disulfide bond formation and protein fold; 2) variants decrease the binding activity of the proteins to calcium ions and then prompt the hydrolysis of FBN2; and 3) variants affect the assembly of various domains and change the spatial structure and intermolecular interaction (Xu et al., 2020). Both the p.C1365R (cysteine changed to arginine) and p.C795F (cysteine changed to phenylalanine) variants were, respectively, located on two cb-EGF-like domains. The two altered amino acid sites were highly conserved throughout evolution (Figure 2A). Three-dimensional modeling of FBN2 in rats confirmed that the Cys residue in these motifs performed an essential role in stabilizing the structure by forming disulfide bonds, and these regions in humans were the same as those in rats (Figures 2B,C). Thus, we speculated that the *FBN2* variants (c.4093T>C, p.C1365R and c.2384G>T, p.C795F) destroyed the disulfide bonds, destabilized the protein structure, affected the functions of the connective tissue, and eventually, led to the occurrence of diseases of multiple organs throughout the body.

Up till now, there have been at least 188 reported variants of *FBN2* (http://www.hgmd.cf.ac.uk/ac/gene.php?gene=FBN2). Most of these variants are associated with CCA and cluster in the cb-EGF-like domains, especially exons 24–33, which is a variant hot spot (Frederic et al., 2009). The p.C1365R variant occurred in exon 31. The missense variants (such as c.1123T>C, p.C375D; c.3769T>C, p.C1257R; c.4151G>T, p.C1384F; and c.4216T>C, p.C1406R) were identified in CCA cases and altered cysteine to other amino acids (AAs), affecting disulfide bond formation (Gupta et al., 2002; Callewaert et al., 2009; Alazami et al., 2015; Liu et al., 2015). In this study, our *FBN2* variants (c.4093T>C, p.C1365R and c.2384G>T, p.C795F) also affected disulfide bond formation in the same way and led to protein misfolding, confirmed by the three-dimensional modeling of rat FBN2.

## Conclusion

In summary, our study reports two novel *FBN2* variants (c.4093T>C, p.C1365R and c.2384G>T, p.C795F), which were identified in two Chinese families with CCA by WES and Sanger sequencing. Based on all the factors, such as patient phenotypes, functional alterations, and *in silico* prediction analyses, the *FBN2* variants were shown to be disease-causing and associated with CCA. Our findings have enriched the spectrum of *FBN2* variants, contributing to the understanding of the correlation between genotypes and phenotypes of CCA, emphasizing the pathogenicity of disulfide bond disruption in FBN2, and serving as a reference in genetic consultation and prenatal diagnosis for CCA.

## Data Availability

The data sets presented in this study can be found in online repositories. The names of the repository/repositories and accession number(s) can be found in the article/Supplementary Material.
